# Interfacial Viscoelastic Moduli of Surfactant- and Nanoparticle-Laden Oil/Water Interfaces Surrounded by a Weak Gel

**DOI:** 10.3390/nano15191489

**Published:** 2025-09-29

**Authors:** Lazhar Benyahia, Ahmad Jaber, Philippe Marchal, Tayssir Hamieh, Thibault Roques-Carmes

**Affiliations:** 1 Institut des Molécules et Matériaux du Mans (IMMM), UMR 6283 CNRS—Le Mans Université, 1, Avenue Olivier Messiaen, 72085 Le Mans, Cedex 9, France; ahmadjaber.795@hotmail.com; 2 Université de Lorraine, CNRS, LRGP, 1, Rue Grandville, 54001 Nancy, France; philippe.marchal@univ-lorraine.fr; 3 Faculty of Science and Engineering, Maastricht University, P.O. Box 616, 6200 MD Maastricht, The Netherlands; t.hamieh@maastrichtuniversity.nl

**Keywords:** Silica-laden O/W interface, Span 80-laden O/W interface, κ-carrageen, interfacial rheology, interfacial viscoelastic moduli, indopol oil, paraffinic oil

## Abstract

This work aims to study the effect of the bulk rheology of a complex system on the apparent interfacial viscoelastic response of a rising oil droplet of a paraffinic oil (Indopol) undergoing sinusoidal volume dilatations insidean aqueous phase containing a hydrogel. The modulation of the interfacial viscoelasticity is obtained using Span 80 surfactant or fumed silica nanoparticles. The rheology of the continuous phase is tuned by adding 3 to 5 g/L of κ-carrageenan (KC) to switch the continuous aqueous phase from a liquid to a gel state at 15 °C. When KC is liquid, the presence of Span 80 or nanoparticles at the liquid/liquid interface increases the apparent interfacial elastic modulus. However, when KC becomes a weak gel, the apparent interfacial elastic modulus depends on the nature of the surface-active agents. Indeed, if the presence of silica hard nanoparticles enhances the apparent elasticity of the interface, adding Span 80 weakens the apparent elasticity of the interface. These trends are discussed in terms of the localization of the deformation and slippage at the interfaces.

## 1. Introduction

The stabilization of emulsions is reached using surface-active species, such as surfactants and nanoparticles. In fact, their adsorption at the liquid/liquid interface prevents the droplet fusion. Surfactants can adsorb to the interface due to their amphiphilic features giving them affinity to both phases. The respective affinity of such molecules for the oil or aqueous phase is related to the so-called hydrophilic–hydrophobic balance (HLB). On the other hand, the amphiphilicity of solid nanoparticles is allowed by the presence of different chemical moieties at their surface. The adsorption ability of nanoparticles and their amphiphilicity is related to the wettability of the particles. The wettability is characterized by the contact angle of the particles at the water/oil interface. This approach is empirical and considers that the phase which preferentially wets the particles is the continuous phase of the emulsion. The more efficient adsorption of the particles occurs for a contact angle of 90°, for which the particles display the higher amphiphilicity. To adsorb to the interface, they must cross an activation energy barrier but, once bypassed, the desorption energy is much higher than k_B_T leading to very stable systems [1,2,3,4]. Note that k_B_ is the Boltzmann constant and T is the temperature.

The adsorption of these surface-active species leads to changes in some emulsions’ features. For instance, the interfacial tension drops with the adsorption of surfactants at the liquid/liquid interface [5,6] while it is not always the case with particles [7,8,9]. Furthermore, a mechanical reinforcement of the interface appears that may end in a solid-like behavior in some cases [10,11,12]. The measurements of these properties (interfacial tension and viscoelasticity) turn out to be very useful and important to understand their effect on the emulsion stability and general behavior [13,14,15,16].

The mechanical properties of the liquid interface can be quantified by interfacial rheology methods in both shear [10,17,18] and dilatational modes [19]. The latter could be, for instance, a Langmuir film balance or the drop shape tensiometry [20,21].

The principle of the droplet-based tensiometry consists of relating the evolution of the interfacial tension to the volume change in the droplet [20]. As the liquid in both the drop and the continuous phase is set in flow, their rheological properties should be considered carefully. In principle, the pendant drop method is well suited to liquids with low viscosities. However, for many emulsions, the continuous phase, and even the suspended phase, may present a large viscosity and even a non-Newtonian behavior [22,23,24,25,26]. Emulsions with a complex continuous phase, such as gel or Pickering systems, are widely used for applications in the field of cosmetics and the food industries.

It therefore seems relevant to evaluate the contribution of a complex rheology of one of the liquid phases on the interfacial viscoelastic moduli measurements. This aspect has rarely been addressed in the literature. To our knowledge, only a few studies were concerned by the surface tension measurements in strong gels [27,28]. For dynamic measurements, it appears that the viscous forces cannot be neglected in dynamic pendant drop analysis [29]. In fact, the shear resistance of the interface can appear at a high frequency during the elongation of the interfacial film [30].

In our previous work [31], we started to tackle this topic by considering the case of a weak gel. Thus, the continuous phase was an aqueous solution of κ-carrageenan (KC). This biopolymer presents a reversible coil–helix transition with a temperature that may lead to gels whose elastic modulus are simply tuned while adjusting the polysaccharide concentration, salt chemistry and ionic strength. In addition, the sol–gel transition presents a hysteresis with the temperature. For a given temperature, the sample can exhibit a gel-like or liquid-like behavior, depending on its thermal history: if it was cooled or heated beforehand. The sol–gel transition can also be conducted at a fixed temperature by changing the KC concentration. At low concentrations (1–3 g/L) at 15 °C, the KC behaves as a liquid, while at higher concentrations (higher than or equal to 5 g/L), the KC is in the form of a gel. The higher the KC concentration, the higher the strength of the gel. This is why we use two KC concentrations of 3 g/L and 5 g/L at 15 °C in the present study. The thermosensitive behavior of the system is used to set up the liquid/liquid system. The KC gel is formed in situ at high temperature, where the KC is liquid (30 °C), in order to have efficient contact between the indopol oil drop and the continuous aqueous KC phase. Then, the temperature is switched to 15 °C in order to obtain a weak KC gel for the higher concentrations of KC.

In addition, the KC is a non-surface-active agent and, thus, no adsorptions of the polymer chains are supposed to occur. Consequently, we were able to demonstrate the direct effect of the rheology of the interfacial continuous phase on the interfacial viscoelastic measurements. In particular, weak gels’ influence upon the apparent viscoelastic moduli of the oil/water interface was considered. The results showed a higher interfacial modulus when the continuous phase became a gel. It is more appropriate to speak about apparent viscoelastic interfacial moduli, since the moduli took into account the effect of the bulk continuous phase. So, it appears relevant to extend our study when surface active species are introduced, namely surfactant (soft) and nanoparticle (hard).

For the first time, the present work aims to study interfacial viscoelasticity (IVE) using a rising droplet of Indopol oil in very weak KC hydrogel in the presence of surfactant or nanoparticles that are adsorbed at the oil/water interface. Indopol is a paraffinic oil. The surface-active species are initially introduced in the Indopol organic phase. Span 80 is used as a surfactant and Aerosil 816 fumed silica as nanoparticles. Span was used as a surfactant due to its affinity for the oil phase and mainly because it is currently used in the cosmetic, pharmaceutic and food industries. Hydrophobized silica nanoparticles were selected as model nanomaterials because this material is currently used in industry to stabilize emulsions and foams. The idea was to work with hard nanoparticles that are able to be dispersed in Indopol oil and also to adsorb at the oil/water interface. The KC concentrations in aqueous solutions are varied from 3 to 5 g/L to switch the continuous phase from a liquid to a gel state at 15 °C, respectively. The apparent interfacial viscoelastic properties are then determined for different situations and analyzed regarding the rheological properties of the continuous phase. These results are discussed within the frame of the potential strain inhomogeneity at the droplet interface.

## 2. Materials and Methods

### 2.1. Chemicals

All ingredients are used as received without any further purification. The oil phase is a polybutene-based chemical purchased from INEOS Oligomers under the trade name Indopol^®^ (Lavera, France). Here, Indopol L-6 was used. It is isobutylene and it was chosen for its low viscosity (9.842 mPa.s at 20 °C), which allows us to avoid the effects of viscosity on oscillations. In addition, Indopol L-6 oil was also used, due to its stable value over time during the interfacial tension measurement at the water/Indopol interface, with the Indopol drop in contact with a water phase.

The model gel used in this work is a κ-carrageenan (KC) aqueous solution. It is a sulfated polysaccharide extracted from seaweed. This biopolymer was chosen for the following reasons. First, its thermo-sensibility and its sol–gel reversibility [32,33,34] were important. Second, the absence of surface activity of KC was an important aspect in order to avoid the adsorption of the gel at the oil/water interface and, consequently, eliminate the direct contribution of the gel to the interfacial viscoelasticity [35,36]. Another argument was the formation of a very weak gel at our concentration of 5 g/L (with an elastic modulus G′ at around 1 Pa). This aspect was important because the Indopol drop had to oscillate inside a medium of moderate viscosity and elasticity without important viscoelastic effects. It was purchased from Sigma-Aldrich (batch #BCBX5072, Darmstadt, Germany). A stock solution of KC was prepared by dissolving the appropriate mass of KC powder in ultra-pure water (18 MΩ.cm) under stirring for 2 h at 80 °C. The homogeneous solutions were allowed to rest for one day in the fridge. Then, the obtained solution was diluted in water to reach KC concentrations of 3 or 5 g/L.

First, the choice of the surfactant can be briefly discussed. In our system, a rising drop of Indopol was formed, surrounded by an aqueous phase containing κ-carrageenan. The surfactant must not be introduced in the continuous aqueous phase in order to avoid any interactions with κ-carrageenan. Consequently, the surfactant had to initially be present in the Indopol organic phase (in the drop). It was necessary to use a surfactant that displayed a better affinity for the oil than for the aqueous phase. Consequently, the selected surfactant may have a Hydrophilic–Lipophilic Balance (HLB) lower than 10. Sorbitan monooleate, known as Span^®^ 80, is selected due to its affinity for the oil phase and mainly because it is currently used in the cosmetic, pharmaceutic and food industries. It is a liquid surfactant purchased from Sigma-Aldrich (batch #BCBZ8539, Darmstadt, Germany). First, a stock solution at 20 wt% of Span 80 in Indopol^®^ was prepared using a vortex. Then, the desired surfactant concentrations were achieved through dilution of the surfactant stock solution by adding the necessary amount of pure oil up to 0.05 and 0.1% wt%. Air bubbles remaining in the solutions were removed using a vacuum desiccator.

Silica nanoparticles were selected as model nanomaterials. This material is currently used in industry to stabilize emulsions and foams. The idea was to work with hard nanoparticles that were able to be dispersed in Indopol oil and also to adsorb at the oil/water interface [37,38]. In this study, we selected hydrophobized silica particles to be dispersed in Indopol in order to have only KC in the aqueous phase. The nanoparticles (NP) used were fumed silica. They are supplied from Evonik, Essen, Germany (batch #150042435), under the trademark Aerosil R816. The primary particles, of about 12 nm, aggregated in a fractal-like structure of few microns and display a BET surface area around 190 ± 20 m^2^/g [24]. They were modified by hexadecyl groups to bring them a partial hydrophobicity feature. The NP were dispersed in Indopol oil up to 0.5 wt%, using an ultrasound probe at 25 kHz and 22% amplitude for 3 min. Note that Isopropanol (IPA, provided by Aldrich, Darmstadt, Germany, 99% pure) is introduced as a co-solvent at 10 wt%.

All the experiments were conducted at 15 °C because the KC solutions behave as a gel at this temperature for the higher concentrations [32,33,34,35,36]. But, by playing with the KC content, it is possible to tune between liquid- and solid-like behavior. Indeed, a KC content of 3 g/L, the KC aqueous solution presented a liquid-like behavior. Conversely, at a KC concentration of 5 g/L, the KC aqueous solution was in the form of a weak gel, i.e., it displayed a solid-like behavior.

### 2.2. Bulk Rheology

Bulk rheology of KC aqueous solutions at 3 g/L and 5 g/L was determined using a stress-controlled rheometer (DHR3; TA Instruments; Guyancour, France). Before loading the sample, the coaxial cylinders (Couette) geometry (rotor radius = 14 mm; stator radius = 15 mm; immersed height = 42 mm) were preheated at 15 °C. Measurement temperature was maintained for at least 1000 s to reach a steady state in the sample before any rheological measurement. The frequency sweep was performed between frequencies f between 1 and 0.01 Hz at 10% strain which was in the linear regime for all samples at 15 °C. In oscillatory rheology, the results are usually expressed as a function of the viscoelastic modulus G*:

G* = G′ + i G″(1)

where the real part G′ is the storage modulus (or elastic modulus) and the imaginary part G″ is the loss modulus (or viscous modulus). These two moduli account for the solid-like and liquid-like character of a viscoelastic material, respectively.

### 2.3. Interfacial Rheology: With a Drop-Profile Tensiometer

Interfacial tension IFT (static) and interfacial viscoelastic dilatational moduli,${\;K}_{s}^{\prime }$ (elastic) and $K_{s}^{\prime \prime }$ (viscous), of net or laden-oil/water interfaces were investigated via a drop-profile tensiometer (TRACKER; Teclis; Civrieux-d’Azergues, France). The picture of the setup is displayed in Figure S1 of the Supporting Information. For the experiments, otherwise specified, a rising drop of Indopol-based solution or suspension (containing surfactants or particles, respectively) was put into contact with the aqueous solutions, filling the quartz transparent cell. An iron-curved needle of internal diameter of 0.84 mm and external diameter of 1.3 mm was used.

For the experiments with KC in the form of a gel (KC concentration of 5 g/L at 15 °C), a special procedure was employed. The thermosensitive behavior of the system was used to set up the liquid/liquid system. The KC gel was formed in situ in order to have an efficient contact between the Indopol drop and the continuous aqueous KC phase. The system was put into contact at a temperature of 30 °C, where the KC was liquid at 5 g/L. Then, the temperature was switched to 15 °C in order to obtain a weak KC gel. For the other configurations (pure water and aqueous solution with a KC concentration of 3 g/L), the temperature was fixed to 15 °C during the whole process.

The time evolution of the interfacial tension was studied by keeping the oil drop volume constant at 10 µL. For the interfacial viscoelasticity measurements, the starting drop volume was fixed to 10 µL. The measurements were conducted after an equilibration time of around 600 s, where a constant value of the interfacial tension was reached (see Section 3.2.1 and Section 3.3.1). After that period, the viscoelasticity of the interface is probed via sinusoidal volume dilatations and compressions. While the experiments of oscillatory droplets involved a volume change, the true parameter to consider is the area change, since the viscoelasticity of the interface is related to the ratio of the variation in the interfacial tension divided by the variation in the natural logarithm of the droplet area [39,40,41]. The elastic/storage interfacial modulus $K_{s}^{\prime }$ and loss/viscous interfacial modulus $K_{s}^{\prime \prime }$ can be obtained from these experiments. The notations $K_{s}^{\prime }$ and $K_{s}^{\prime \prime }$ are reported here for dilatational apparent elastic and viscous modulus, respectively. It is also usual to use E’ and E’’ as notations instead of $K_{s}^{\prime }$ and $K_{s}^{\prime \prime }$ in the literature. Here, the symbols $K_{s}^{\prime }$ and $K_{s}^{\prime \prime }$ were adapted to highlight the presence of a weak gel in the continuous phase [31]. The amplitude of volume variation (sinusoidal volume dilatation) was set to 10% of the initial drop volume, which we verified was in the linear regime of the rheological behavior (Figures S2 and S3 of the Supporting Information). The oscillation frequency (f) varied from 0.05 to 1 Hz. Precisely, at each frequency, a sequence of five oscillations were applied, followed by 30 s of rest. This sequence is repeated until a steady state of the moduli was obtained. The value of $K_{s}^{\prime }$ and $K_{s}^{\prime \prime }$ reported in the figures was the average of the final series of 5 oscillations. We know that at a frequency of 1 Hz, the system was close to the hydrodynamic limit of oscillatory experiments [42,43,44]. However, the exact value of the frequency limit depends significantly on the viscosity of the two fluids. The data at a frequency of 1 Hz are consistent with the rest of the experimental points at lower frequencies. For these reasons, the data at a frequency of 1 Hz are displayed in the following figures. All measurements were repeated at least three times, ensuring that the variability of the results was less than 10%. Before the measurements, the equipment was preheated at 15 °C and maintained during all the measurements. For the special cases, for which the elastic and viscous interfacial moduli are studied as a function of time (see Section 3.2.1 and Section 3.3.1,), a different procedure was used. No equilibration time was used. The first measurement was conducted a few seconds after the droplet formation, via a sequence of five oscillations. Then, the drop was maintained as immobile. After a given time, a new set of droplet oscillations was performed. These experiments were conducted to fix the equilibration time for the other experiments.

## 3. Results

### 3.1. Bulk Properties of KC Solutions

**Figure 1:**
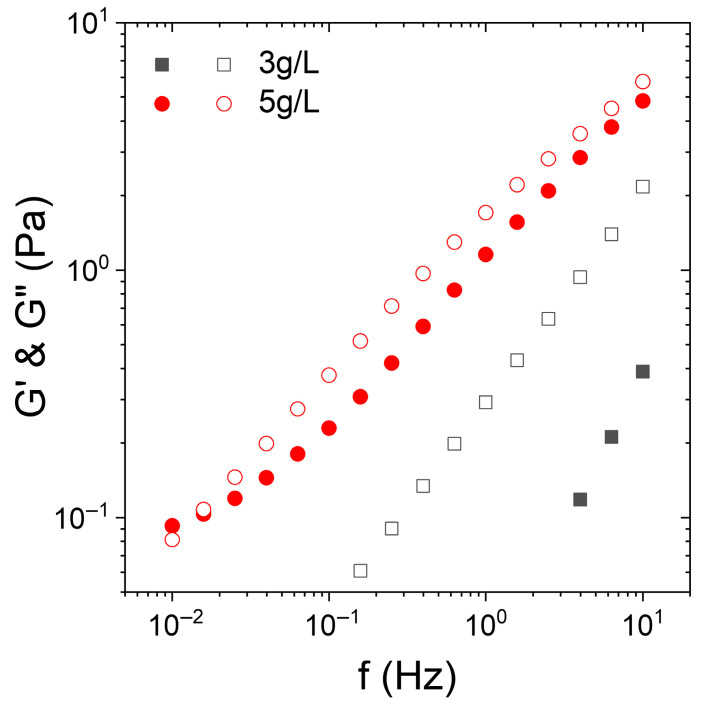
Frequency dependence of the elastic (G′, closed symbols) and the viscous (G″, open symbols) modulus of KC aqueous solutions at 15 °C containing KC at 3 g/L (squares) and 5 g/L (circles).

First, the rheological behavior of KC solutions at 15 °C is presented. For this purpose, the elastic (G′) and the viscous (G″) moduli are plotted as a function of the frequency (f) for aqueous solutions containing KC at 3 and 5 g/L in Figure 1.

At a concentration of KC of 3 g/L, G′ is almost undetectable at low frequencies and displays a slope close to two (in log/log scale) while G″ versus the frequency exhibits a slope of 1. At this concentration, the KC solution presents a liquid-like behavior. Conversely, for a KC concentration of 5 g/L, a different behavior is observed. G′ becomes detectable and significant and almost equal to G″. Both G′ and G″ present a power law dependence with the frequency with an exponent around 0.6. This indicates that the KC solution goes through a percolation structure, leading to a gel-like behavior. More precisely, the trend of G′ and G″ vs. the frequency is a signature of a weak gel [45,46,47,48]. To confirm this trend, we refer to the articles by Winter and Chambon on the determination of the gel point (Winter–Chambon criterion) [46]. Their work shows that at the gel point, tan delta = G″/G′ is independent of the oscillation frequency with G″ > G′, i.e., delta > 45°. This independence of tan delta with respect to the frequency implies that the ratio G″/G′ remains constant over the explored frequency range, which results in a power law spectrum, i.e., two parallel lines in log-log coordinates as on the graph displayed in Figure 1. Moreover, Winter and Chambon showed that the exponent (i.e., the slope in log-log) was typically 0.7 in this zone, which seems to be the case on our graph [46,47].

### 3.2. Surfactant-Laden Interfaces

#### 3.2.1. Kinetic of Surfactant Adsorption at O/W Interface

**Figure 2:**
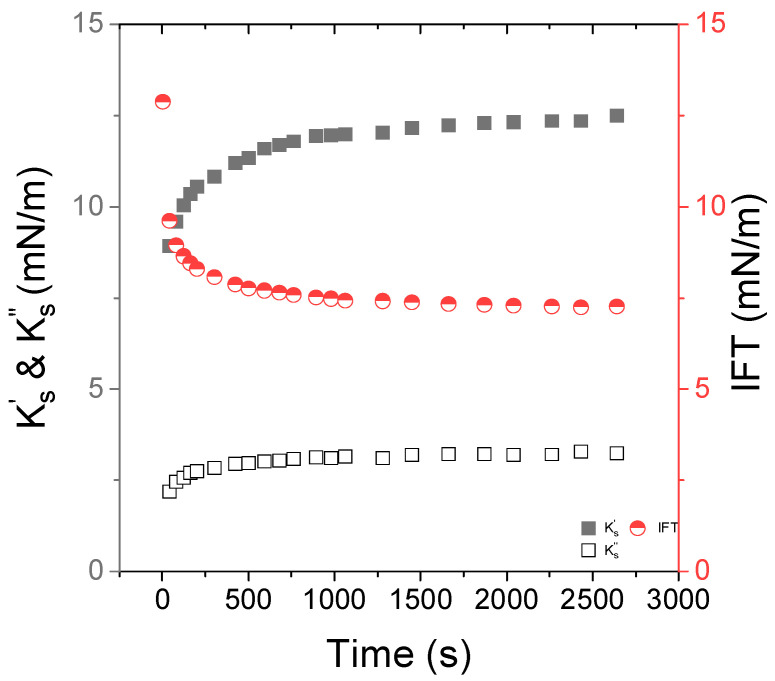
Time dependence of the interfacial viscoelastic moduli,${\;K}_{s}^{\prime }$ (closed squares) and ${\;K}_{s}^{\prime \prime }$ (open squares), at f = 0.5 Hz and the interfacial tension IFT (circles) of the Indopol/water interface in the presence of 0.05 wt% of Span 80. The temperature is set to 15 °C.

First, we reported the interfacial tension (IFT) and interfacial viscoelastic properties of surfactant-laden O/W interface in pure water. Figure 2 represents the time evolution of IFT,${\;K}_{s}^{\prime }$ and ${\;K}_{s}^{\prime \prime }$ in the presence of 0.05 wt% of Span. The temperature was set to 15 °C.

The interfacial tension of a pure Indopol/water interface was 18 mN/m (see Figure S4 of the Supporting Information). In the presence of Span, the interfacial tension decreased first sharply and, then, tended slowly towards a plateau at 7–8 mN/m, reflecting the relative slow kinetic of Span adsorption at the O/W interface. The interfacial tension was of a similar order of magnitude to what was reported for Span 80 in paraffin oil [15]. A similar trend is observed for the interfacial viscoelastic moduli but, the latter increased from 9 to 12 mN/m, and from 2 to 3 mN/m for ${\;K}_{s}^{\prime }$ and ${\;K}_{s}^{\prime \prime }$, respectively. At a frequency of 0.5 Hz, the Span-laden interface presented rather an elastic response. It appears that the equilibrium state was mainly reached after 600 s. Thus, for all the other experiments, a waiting time of at least about 600 s was systematically kept.

These kinetics were not significantly affected by the Span concentration (see Figure S4 of the Supporting Information), even though the interfacial tension decreased gradually with Span concentration up to 4.78 mN/m at 0.1 wt%. Above this concentration, the oil drop started to detach, preventing further reliable measurements. In the following figure, Span concentrations were limited to 0.05 and 0.1 wt% since they lead to roughly similar interfacial tensions and, thus, similar surface coverages.

#### 3.2.2. Interfacial Viscoelasticity of Span-Laden O/KC-W Interfaces

**Figure 3:**
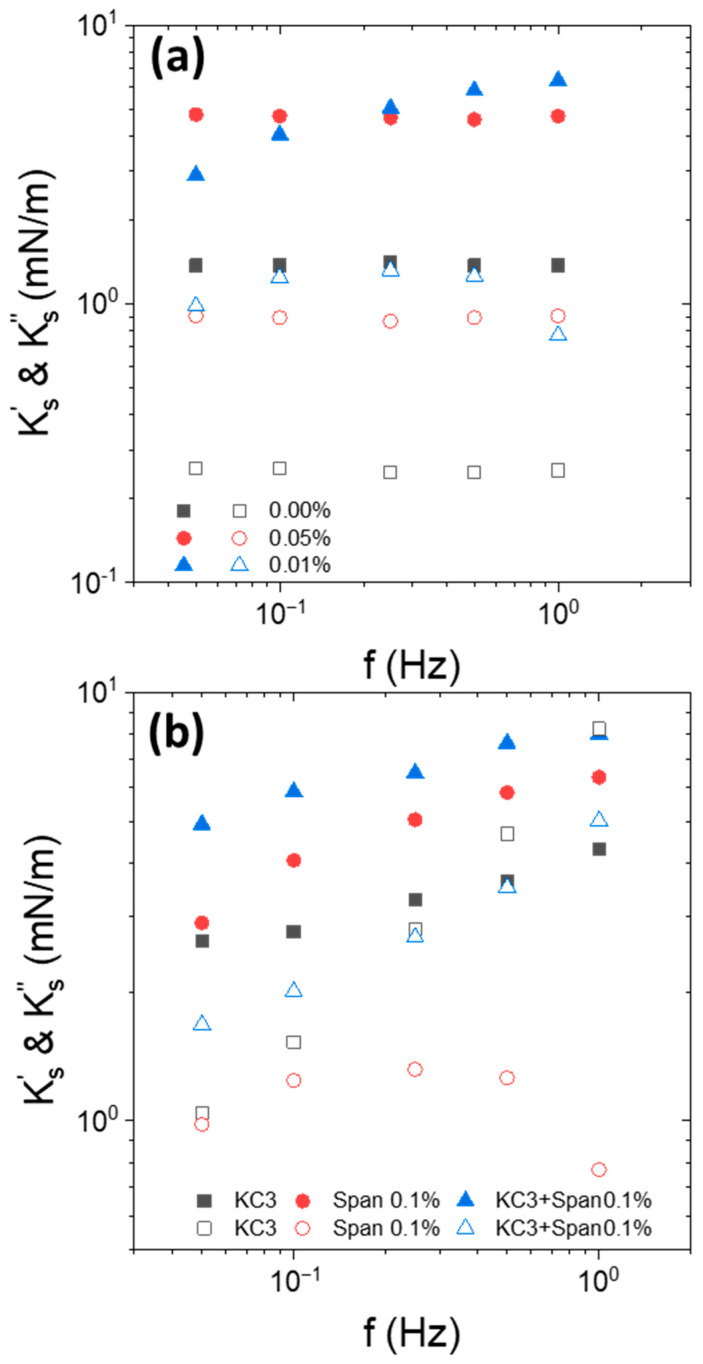
Frequency dependence of interfacial elastic moduli ${\;K}_{s}^{\prime }$(closed symbols) and viscous moduli ${\;K}_{s}^{\prime \prime }$ (open symbols) of (**a**) Indopol/water interface with and without Span, as indicted in the figure, at 15 °C, (**b**) Indopol/water-KC interface with and without Span at 15 °C and 3 g/L of KC. Span concentration is equal to 0.1 wt%. “KC3”corresponds to the data in the presence of KC but in the absence of Span. “Span” corresponds to the data without KC and in the presence of Span.

The KC free Indopol/water (O/W) interface in the presence and absence of Span was considered. The apparent interfacial viscoelastic moduli at 15 °C are shown in Figure 3a.

For net or Span-laden O/W interface, ${\;K}_{s}^{\prime }$ was larger than ${\;K}_{s}^{\prime \prime }$ and they were quasi-independent of the frequency. However, in the presence of Span, the moduli increased significantly compared to that of the net interface. This increase was most likely caused by the adsorption of Span molecules to the O/W interface. Nevertheless, increasing Span concentration from 0.05 to 0.1 wt% was rather non-significant on the moduli’s increase. The frequency dependence of the moduli, as well as their order of magnitude, were similar to those classically encountered with surfactants at liquid/liquid interfaces [49,50].

The second time, we were interested by the interfacial viscoelastic response in the presence of Span-based oil droplet suspended in an aqueous KC solution at 3 g/L (Figure 3b). We recalled that KC solutions presented a liquid-like behavior at 3 g/L of KC.

In the presence of liquid KC, the interfacial elastic moduli were larger in the presence of Span than with pure oil. This confirms that the adsorption of Span at the O/W interface increased the elasticity of the interface. It is also interesting to note that the elasticity of Span-Indopol/KC-water interface was similar to that of Span-Indopol/water interface without KC. The shift of around 1–2 mN/m was in the range of experimental uncertainties. This indicated that the apparent elasticity was driven by the adsorption of the Span surfactant at the interface.

**Figure 4:**
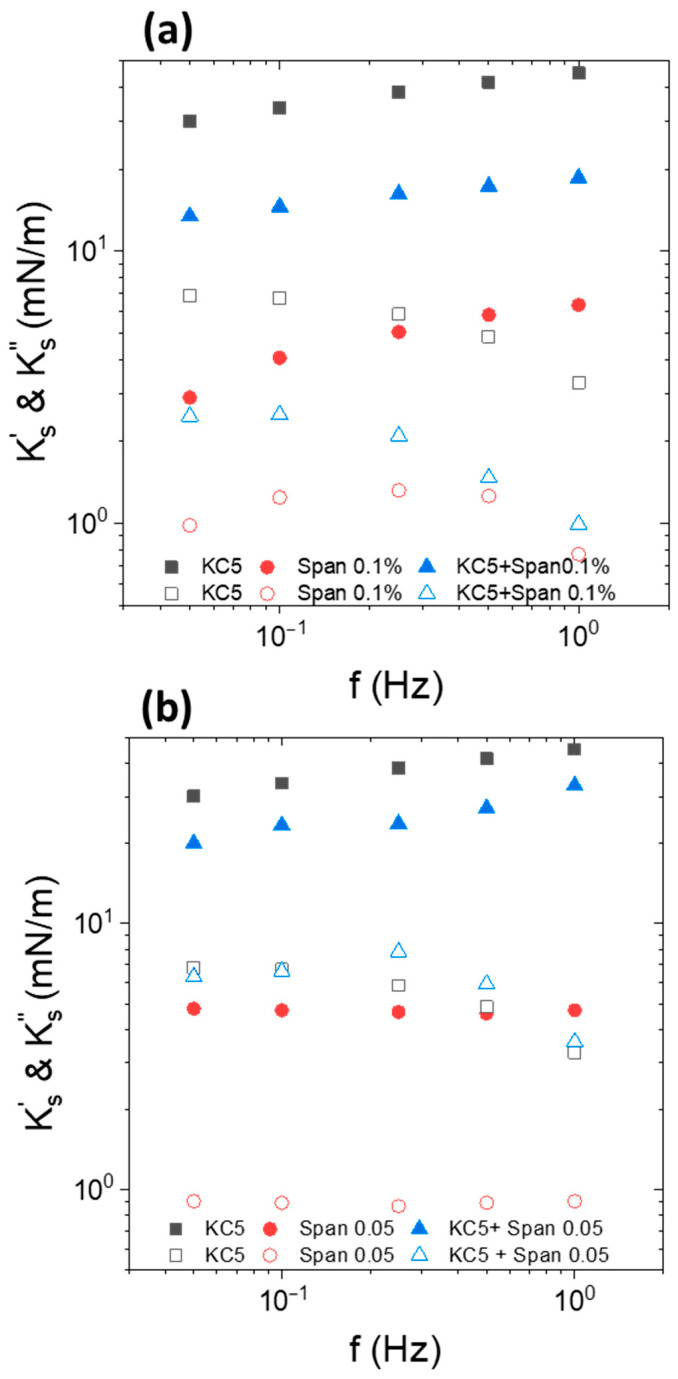
Frequency dependence of interfacial elastic moduli ($K_{s}^{\prime }$(closed symbols) and viscous ${\;K}_{s}^{\prime \prime }$ (open symbols)) of Indopol/water-KC interface without and in the presence of Span at (**a**) 0.05 wt% and KC at 5 g/L, and (**b**) 0.1 wt% and 5 g/L of KC. “KC5”corresponds to the data in the presence of KC but in the absence of Span. “Span” corresponds to the data without KC and in the presence of Span.

Figure 4a,b represent the evolution of the apparent elastic and viscous moduli with the frequency in the simultaneous presence of Span and 5 g/L of KC. Note also that the data without KC and only Span are added in the figures.

For 5 g/L of KC, the continuous phase was in the form of a weak gel. In that configuration, higher interfacial elastic moduli were obtained in the presence of KC but in the absence of Span. More particularly, the apparent elastic moduli follow the order of ${\;K}_{s}^{\prime }$ (KC) > ${\;K}_{s}^{\prime }$ (KC + Span) > ${\;K}_{s}^{\prime }$ (Span). These trends were observed with the two surfactant concentrations. This confirms that the observed effect was not an artifact and has a real physical meaning. Another interesting feature is that the frequency dependence of the apparent interfacial elastic modulus for KC + Span system (“KC5 + Span”) falls between KC alone (“KC5”) and Span alone (“Span”) curves. In other words, the apparent interfacial elasticity measured was lower in the presence of Span when the continuous phase was a weak gel. For the gel-like KC fluids, adding Span contributed to the decrease in the interfacial viscoelasticity moduli of the interface. This emphasizes that the effect of the continuous phase in the form of a gel has a significant impact on the apparent viscoelastic trend of the interface.

Some reduction in the dilatational elastic modulus ${\;K}_{s}^{\prime }$ in the presence of surfactants was previously reported in the literature [50,51,52,53]. This occurs at high surfactants concentrations in comparison to low surfactant concentrations. The observed trend for the dilational viscoelasticity arises from the simultaneous and combined effects of (i) interactions between surfactant molecules at the interface and (ii) surfactant molecular exchange between bulk and interface. This drop in elasticity is attributed to the fact that surfactant diffusional exchange between the bulk and the interface becomes prevalent, reducing the interfacial viscoelasticity through the quick replacement of surfactant to the area depleted owing to surface expansion. In the present study, the reduction in $\;{\;K}_{s}^{\prime }\;$ cannot be attributed to the surfactant diffusional exchange between the bulk and the interface due to the low surfactant concentration used here [50,51,52,53]. In addition, the viscoelasticity of the aqueous phase due to the presence of the weak gel of KC also hinders the possible surfactant diffusional exchange between the bulk and the interface.

### 3.3. Nanoparticles-Laden Interfaces

#### 3.3.1. Kinetic of Nanoparticles Adsorption at O/W Interface

The kinetics of NP adsorption at O/W interface is first discussed through the analysis of the time dependence of the interfacial tension and interfacial viscoelastic properties in the case of pure water.

Measuring the interfacial tension or the interfacial viscoelastic moduli of solid particle-laden interfaces is not trivial. Indeed, the diffusion of particles and their entrapment at the interface are not guaranteed, since the diffusion of particles can be drastically slowed with the increase in their size as well as the viscosity of the medium [54,55,56]. Furthermore, particle adsorption requires to overcome an energy barrier to reach the interface [54,55,56,57,58,59,60].

**Figure 5:**
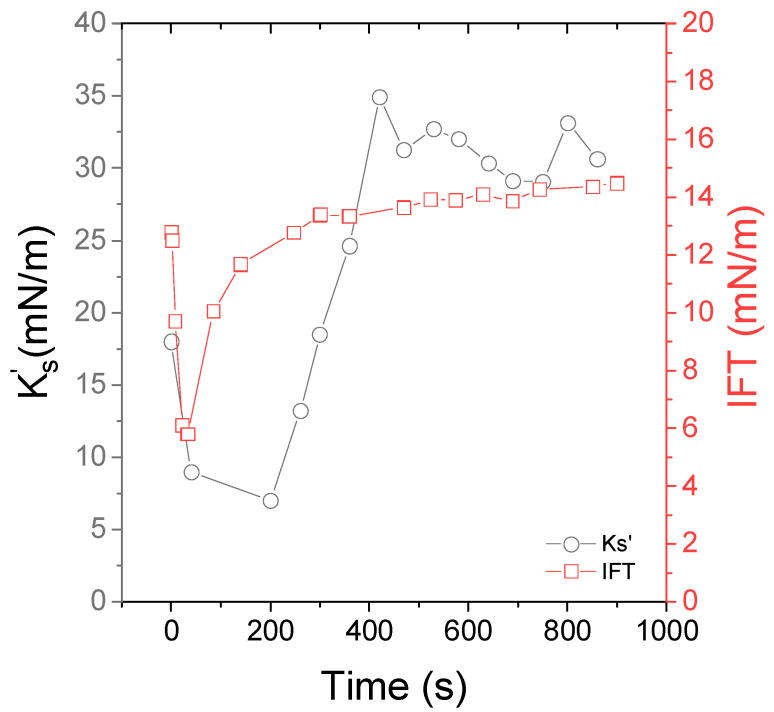
Time evolution of the interfacial tension IFT (red squares) and the interfacial viscoelastic moduli ${\;K}_{s}^{\prime }$ (black circles) of Indopol/water interface in the presence of 0.5 wt% of Aerosil R816 and 10% of IPA. The temperature is set to 15 °C and the frequency to 0.5 Hz.

To overcome this difficulty, a co-solvent, isopropanol (IPA), was added in the oil phase. Figure 5 shows the time dependence of interfacial tension and interfacial viscoelastic moduli of the Indopol/water interface in the presence of Aerosil silica R816 and IPA. First,${\;K}_{s}^{\prime }$ drops and then increases slowly to reach a final plateau after around 400 s. The oil/water O/W interfacial tension follows the same trend than ${\;K}_{s}^{\prime }$. It is worth noticing that the data of Figure 5 mimic the time dependence of the interfacial tension of the Indopol-IPA mixture in contact with water (see Figure S5 in Supporting Information).

In contact with the interface, IPA diffused in water and lowered the interfacial tension. After 50 s, the interfacial tension rose again. We believe that this behavior is related to the evolution of the IPA concentration in the two phases. After about 400 s, the interfacial tension tended towards a value of about 15–16 mN/m, which is slightly lower than the value of the Indopol/water interface (18 mN/m). However, this confirms the absence of isopropanol at the interface after this time. Actually, the isopropanol crossed the liquid/liquid interface to reach the aqueous continuous phase, where it is soluble. In the continuous phase, the evaporation of the isopropanol took place due to the low vapor pressure of this component. In the presence of NPs, the interfacial tension was roughly equal to that of the bare interface in the absence of particles. This does not mean that the particles were not adsorbed, since a lot of studies emphasized that the adsorption of particles does not necessarily modify the interfacial tension [8,9,61].

In the presence of IPA, the particle-laden O/W interface presented a higher elastic modulus of about 30–35 mN/m after 400 s. We recalled that ${\;K}_{s}^{\prime }$ at the free Indopol/water interface is equal to 1–2 mN/m. In the presence of particles, the interfacial elastic modulus reached values around 30–35 mN/m. The order of magnitude was similar to those classically reported in the literature with solid nanoparticles [61,62,63]. This is the proof that IPA helped to decrease the energy barrier for NP adsorption that allowed the latter to be entrapped at the O/W interface under self-diffusion.

As for the Span case, after the two liquids were in contact, at least 600 s was observed before performing interfacial measurements.

#### 3.3.2. Interfacial Viscoelasticity of NP-Laden O/KC-W Interfaces

**Figure 6:**
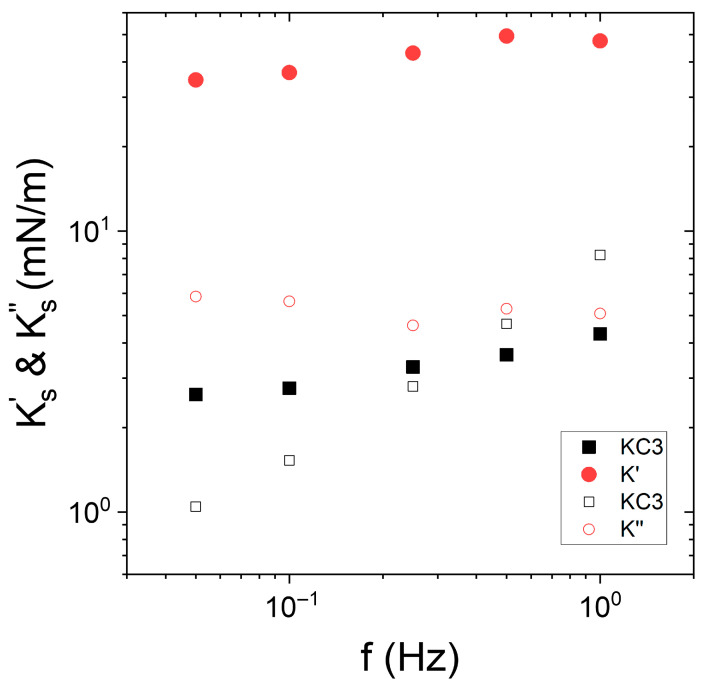
Frequency dependence of interfacial viscoelastic moduli of Indopol-IPA/water-KC interface with (circles, ${\;K}_{s}^{\prime }$, ${\;K}_{s}^{\prime \prime }$) and without (squares, “KC3”) NPs. The close symbols represent the elastic modulus ${\;K}_{s}^{\prime }$ while the open symbols correspond to the viscous modulus ${\;K}_{s}^{\prime \prime }$. The KC concentration is equal to 3 g/L. The silica concentration in oil is fixed to 0.5 wt%. The temperature is 15 °C.

In this part, interfacial rheology experiments were conducted with an Indopol droplet loaded with 10% IPA and containing silica NPs, and oscillated in water medium containing KC at a concentration of 3 g/L. The silica concentration in oil was fixed to 0.5 wt% while the temperature was equal to 15 °C. The results are displayed in Figure 6. For the sake of comparison, the data with the same system but without NP are reported in the same figure. Note that the KC is liquid at KC concentration of 3 g/L.

The interfacial elastic and viscous moduli and their evolution with the frequency depended on the presence or absence of particles. In the presence of silica NPs, the interfacial elastic modulus was significantly larger than the viscous modulus. In terms of frequency dependence,${\;K}_{s}^{\prime }$ did not vary substantially with the frequency, while ${\;K}_{s}^{\prime \prime }$ slightly increases. The value of the interfacial elastic modulus was significantly larger in the presence of silica at the interface. The difference was around one order of magnitude since we reached 3–4 mN/m against 35–50 mN/m in the absence and in the presence of NPs, respectively. This highlights the effect of the enhancement of the interfacial elasticity in the presence of adsorbed particles. This aspect has already been reported in several instances [62,63,64,65,66,67]. Conversely, the frequency dependence of the viscous modulus was not similar in the presence and in the absence of NP. In the absence of silica NP, the viscous modulus increased sharply with the frequency. The adsorption of particles reduced the slope of the ${\;K}_{s}^{\prime \prime }$ vs. f curves, since ${\;K}_{s}^{\prime \prime }$ increased only slightly with the frequency. It also appears that the viscous modulus was higher in the presence of NPs in the range of frequency up to 0.5 Hz. At 0.5 Hz, the two moduli reached approximately the same value.

**Figure 7:**
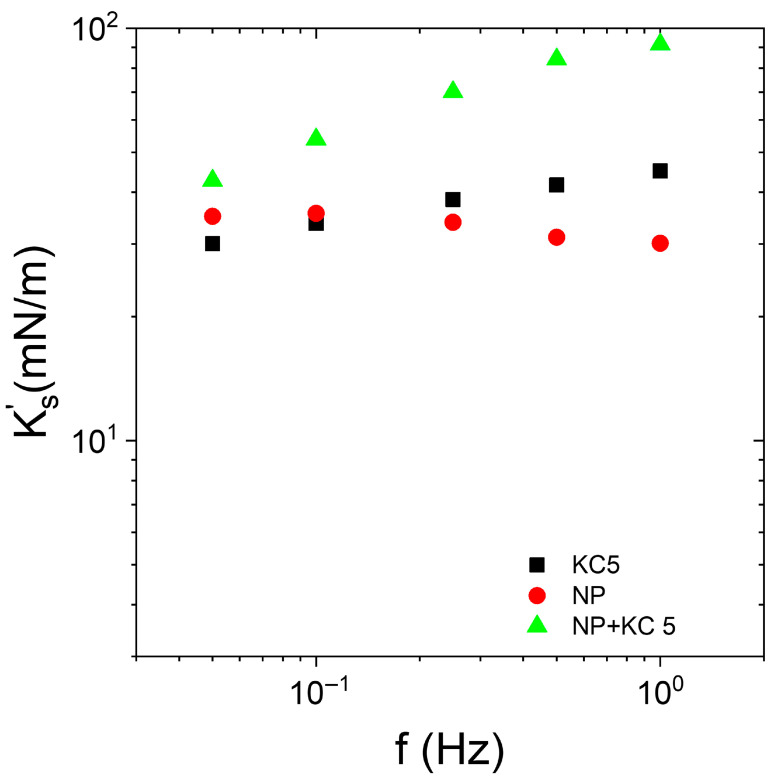
Frequency dependence of interfacial elastic modulus $\;{\;K}_{s}^{\prime }$ of Indopol-IPA/water-KC with (“NP + KC 5”) and without (“KC5”) NPs and in the presence of 5 g/L of KC. “KC5” corresponds to the data in the presence of KC but in the absence of silica. “NP” corresponds to the data in the presence of silica but in the absence of KC. The silica concentration is equal to 0.5 wt%. The temperature is 15 °C.

Interfacial rheology experiments were performed with Indopol-IPA containing silica NPs. The silica concentration in oil was fixed to 0.5 wt%. The oil droplet oscillated in the water medium, which contained KC at a concentration of 5 g/L. During the experiments, the temperature was maintained at 15 °C. The data are reported in Figure 7.

At 15 °C and 5 g/L of KC, the KC formed a weak gel in the aqueous continuous phase. It appeared relevant to compare the impact of the presence or absence of particles on the interfacial moduli. The presence of silica does not modify the shape of the curves ($K_{s}^{\prime }$ vs. frequency). This indicates that the frequency trend of the modulus is driven by the gel continuous phase rather than the layer of particles when the continuous phase is in the form of a gel. Moreover, the presence of the adsorbed layer of particles enhanced the elastic character of the interface, since the ${\;K}_{s}^{\prime }$ values with silica (“NP + KC5”) are larger than those in the absence of particles (“KC5”). This emphasizes that the interfacial layer of particles improved the elasticity of the interface and the system. It can be considered that the apparent interfacial elastic modulus in the presence of silica and gel of KC was a combination of the contribution of the elastic modulus of the particles at the interface and the contribution of the gel continuous phase. The interaction between a hard silica interface layer and a hard gel in the continuous phase strengthened the elasticity of the system. It is also interesting to note that the elasticity of the bare interface with KC (“KC5”) was similar to the interface covered with silica but in the absence of KC (“NP”).

## 4. Discussion

In the framework of this investigation, the surfactant and particles’ effect on the interfacial viscoelastic properties of O/W interface is addressed. In particular, the impact of the rheology of aqueous surrounding medium is investigated. The latter is a KC solution that behaves like a liquid or a weak gel when the KC concentration is lower or higher, respectively. Moreover, KC is known to not be a surface-active agent [68,69]. The lack of interfacial activity at the water/Indopol interface is displayed in Figure S6 in the Supporting Information. The Indopol/water interfacial tension is reported in the presence of KC at different concentrations from 0 to 6 g/L. No difference in the interfacial tension can be measured at the Indopol/water interface in the absence and the presence of KC. These data highlight the absence of interfacial activity of the KC. Consequently, it becomes possible to judge the effect of the complex rheology of the continuous phase of the apparent viscoelastic response of the interface.

First, we ensure that Span and NPs lead to an elastic response during dynamic oscillations of the droplet. It is possible to form an NPs layer if IPA is added. The latter diminishes the adsorption barrier of the particles and allows us to form an elastic NP-based layer at the O/W interface.

When the KC is liquid, the presence of Span and NPs at the liquid/liquid interface increases the apparent interfacial elastic modulus. However, the value of ${\;K}_{s}^{\prime }$ in the presence of particles is substantially larger than that with Span, meaning that the hard-interfacial layer leads to higher apparent interfacial elasticity than with a soft interfacial layer. A similar conclusion was already reported [10,20].

When the KC becomes a weak gel, the apparent interfacial elastic modulus remains the highest with a silica NP layer. More interestingly, when compared with the bare interface (without NP or surfactant), two trends can be extracted. The presence of hard silica NPs at the interface enhances the apparent interfacial elasticity (${\;K}_{s}^{\prime }$ (NP + KC) > ${\;K}_{s}^{\prime }$ (KC)). Conversely, the presence of soft layer made by Span weakens the apparent interfacial elasticity. In other words,${\;K}_{s}^{\prime }$ (Span + KC) is lower than ${\;K}_{s}^{\prime }$ (KC).

The weakened interfacial viscoelastic moduli in the presence of Span when the continuous phase is in the form of a weak gel is an intriguing trend. To our knowledge, this is the first time this phenomenon has been described. A tentative to understand this feature starts from considering the nature of the adsorbed surfactant layer, which is not rigid but rather soft. The final elasticity seems to depend on the interaction between the soft layer of surfactant at the interface and the hard gel of the continuous phase. The gel in the continuous phase seems to fragilize the interface with Span. One possible interpretation is slippage between the surfactant layer and the hydrogel, which leads to strain-like banding. When the drop oscillates, strain-like bands are created, and the hard gel shears the interface. Consequently, the drop deforms more easily. The Span layer acts as a lubricant on the interface between the drop and the bulk. The system deforms where it is easier, then, at the interface. It is reported in the literature that yield stress fluids exhibit such behavior in interfacial rheology experiments [19,28]. It is possible that the dilatation of the interface is opposed by the elastic deformation of the continuous phase, weakening the combined effect of the surfactant layer and the surrounding gel. Numerical simulations would be needed to evaluate such a scenario. This could be an interesting perspective in the future, as to our knowledge, no model or work in the literature has treated this scenario.

## 5. Conclusions

This work investigates the effect of surface-active species on the O/W interfacial rheology in the case of a complex continuous aqueous phase going from liquid to gel state. In particular, the bulk rheology of the latter was tuned using 3 and 5 g/L of κ-carrageenan hydrogel. The interface was covered by a surfactant Span 80 or silica NPs. The apparent interfacial viscoelastic properties were probed at different frequencies using an oscillating rising Indopol-based oil droplet.

When the continuous phase was liquid-like, the apparent elastic modulus increased in the presence of Span 80 and NPs, but much further in the latter case. In the case where the surrounding medium was weak and gel-like, the situation depended on the softness of the interfacial layer. With a hard layer, made with silica NPs, the apparent elastic modulus increased in the presence of KC gel. However, in the case of soft interface, made with a Span layer, the apparent interfacial elastic modulus decreased when the droplet oscillated in the KC gel. It read that a strain-like band phenomenon occurred in this case, implying that the surfactant layer acted as a lubricant and diminished the apparent value of the interfacial modulus.

In perspective, shear interfacial rheology could be helpful to cover all modes of deformation and widen the scope of this research. Modeling could be a useful tool to compare the theoretical scenario with the experimental results, as well as generalize the study.

Concerning the practical benefit of this study, there is great interest in the control of emulsions with a complex continuous phase such as gel or Pickering systems, since they are widely used for applications in cosmetics and the food industry. Our study allows us to demonstrate an extremely important bias for these kinds of emulsions, which will help them to predict what will happen during the manufacture and stabilization of emulsions in industrial fields such as cosmetics and the food industry.

## Data Availability

The data that underline the results that are presented in this paper are not publicly available at this time but can be obtained from the authors upon reasonable request.

## Supplementary Materials

The following supporting information can be downloaded at: https://www.mdpi.com/article/10.3390/nano15191489/s1, Figure S1. (a,b) Pictures of the drop-profile tensiometer (TRACKER) system. (c) Picture of the rising drop. Figure S2. Strain amplitude dependence of interfacial elastic moduli ($K_{s}^{\prime }$ (closed symbols) and viscous $K_{s}^{\prime \prime }$ (open symbols)) of Indopol/water-KC interface containing 5 g/L of KC at 15 °C. The frequency f is fixed to 0.5 Hz. Figure S3. Strain amplitude dependence of interfacial elastic moduli ($K_{s}^{\prime }$ (closed symbols) and viscous ${\;K}_{s}^{\prime \prime }$ (open symbols)) of Indopol/water-KC interface containing 3 g/L of KC at 15 °C. The frequency f is fixed to 0.5 Hz. Figure S4. Time dependence of the interfacial tension Γ of the Indopol/water interface in the presence of Span at different concentrations as indicated in the figure. The notation O/W indicates Indopol/water interface in the absence of Span. Figure S5. Time dependence of interfacial tension Γ of the Indopol/water interface with the addition of 10 wt% of Isopropanol. Figure S6. Indopol/water interfacial tension in the presence of KC at different concentrations at 15 °C.
