# Supplementary material for: Interfacial Viscoelastic Moduli of Surfactant- and Nanoparticle-Laden Oil/Water Interfaces Surrounded by a Weak Gel
Source: Nanomaterials (Basel). 2025 Sep 29;15(19):1489. doi: 10.3390/nano15191489 (PMC12525980; doi:10.3390/nano15191489)
Supplement: Supplementary file 1 [file nanomaterials-15-01489-s001.zip › nanomaterials-3736668-supplementary.pdf]

nanomaterials

Article

Interfacial viscoelastic moduli of surfactant- and nanoparticle-laden oil/water interfaces surrounded by a weak gel

[1] Institut des Molécules et Matériaux du Mans (IMMM), UMR 6283 CNRS—Le Mans Université, 1, Avenue Olivier Messiaen, 72085 Le Mans, Cedex 9, France; ahmadjaber.795@hotmail.com
[2] Université de Lorraine, CNRS, LRGP, 1, Rue Grandville, 54001 Nancy, France; philippe.marchal@univ-lorraine.fr
[3] Faculty of Science and Engineering, Maastricht University, P.O. Box 616, 6200 MD Maastricht, The Netherlands; t.hamieh@maastrichtuniversity.nl
* Correspondence: lazhar.benyahia@univ-lemans.fr (L.B.); thibault.roques-carmes@univ-lorraine.fr (T.R.-C.)

Academic Editor: Rajinder Pal

Received: 18 June 2025
Revised: 10 September 2025
Accepted: 22 September 2025
Published: 29 September 2025

Citation: Benyahia, L.; Jaber, A.; Marchal, P.; Hamieh, T.; Roques-Carmes, T. Interfacial Viscoelastic Moduli of Surfactant- and Nanoparticle-Laden Oil/Water Interfaces Surrounded by a Weak Gel. *Nanomaterials* **2025**, *15*, x. https://doi.org/10.3390/xxxxx

Copyright: © 2025 by the authors. Submitted for possible open access publication under the terms and conditions of the Creative Commons Attribution (CC BY) license (https://creativecommons.org/licenses/by/4.0/).

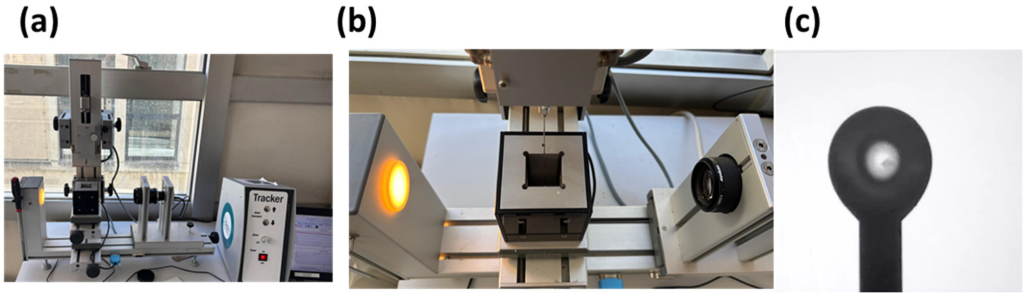

Figure S1. (a,b) Pictures of the drop-profile tensiometer (TRACKER) system. (c) Picture of the rising drop.

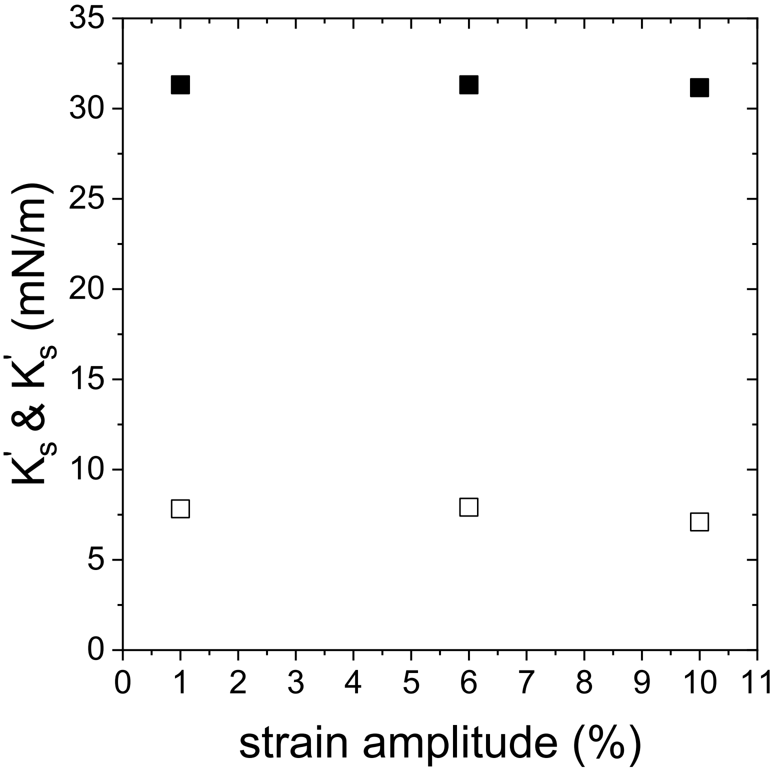

Figure S2. Strain amplitude dependence of interfacial elastic moduli (K_s' (closed symbols) and viscous K_s'' (open symbols)) of Indopol/water-KC interface containing 5 g/L of KC at 15 °C. The frequency f is fixed to 0.5 Hz.

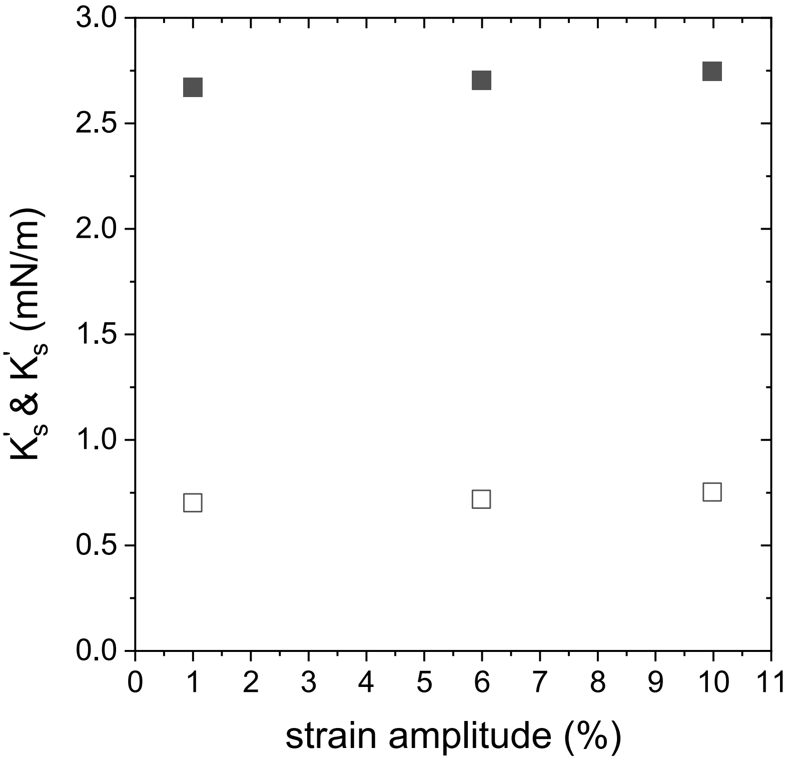

Figure S3. Strain amplitude dependence of interfacial elastic moduli (K_s' (closed symbols) and viscous K_s'' (open symbols)) of Indopol/water-KC interface containing 3 g/L of KC at 15 °C. The frequency f is fixed to 0.5 Hz.

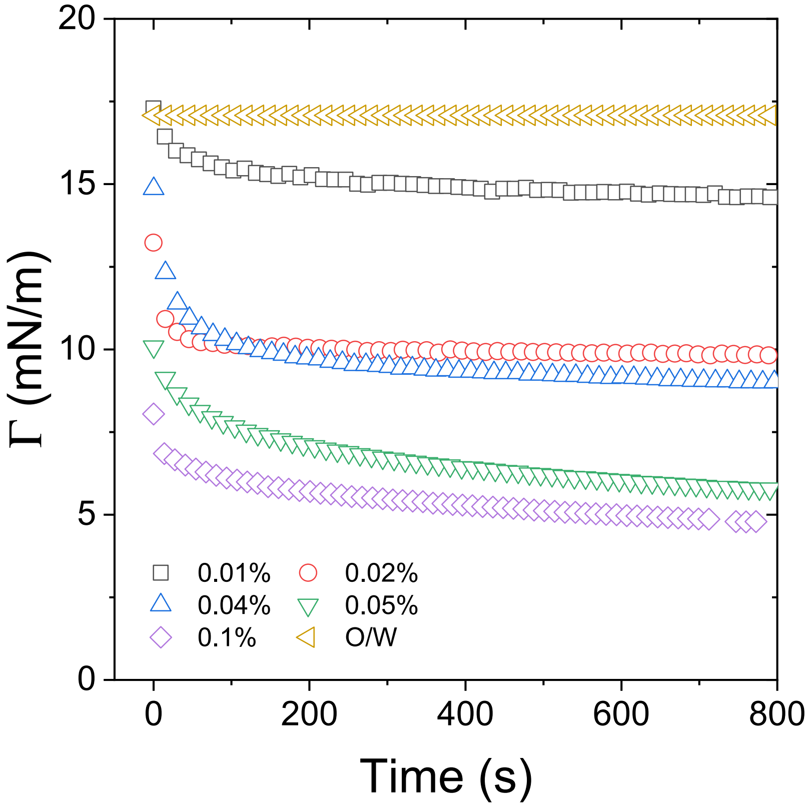

Figure S4. Time dependence of the interfacial tension Γ of the Indopol/water interface in the presence of Span at different concentrations as indicated in the figure. The notation O/W indicates Indopol/water interface in the absence of Span.

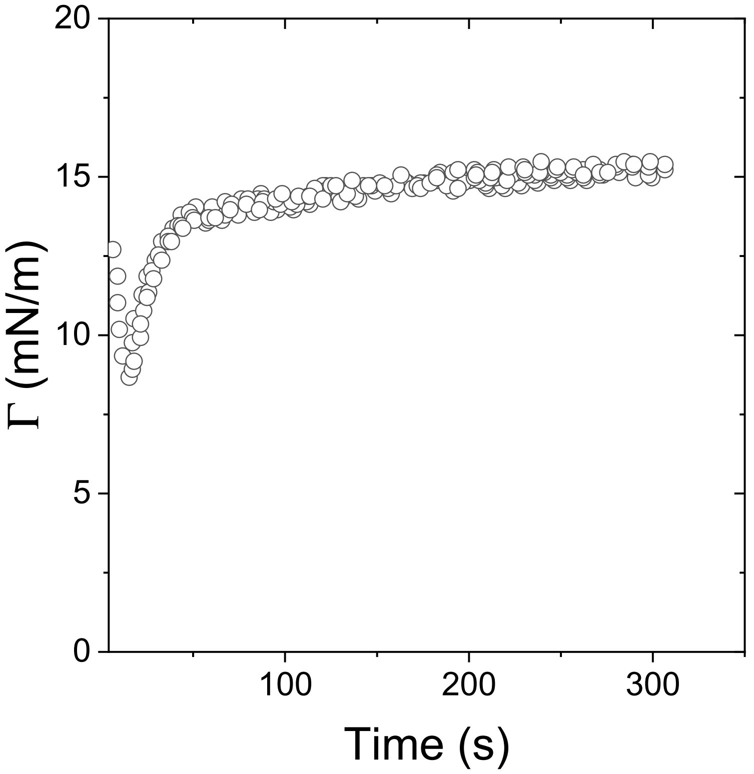

Figure S5. Time dependence of interfacial tension Γ of the Indopol/water interface with the addition of 10 wt% of Isopropanol.

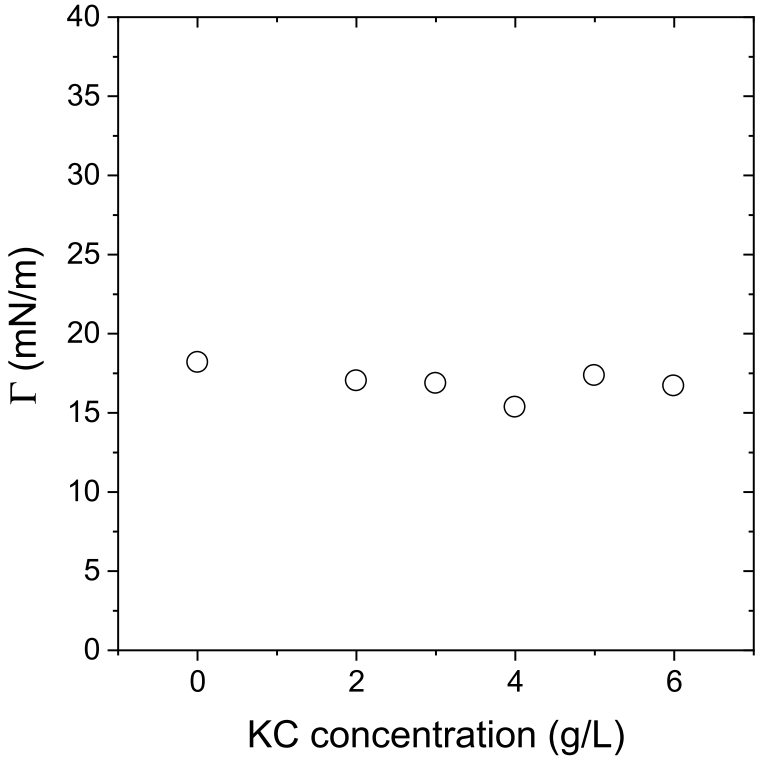

Figure S6. Indopol/water interfacial tension in the presence of KC at different concentrations at 15 °C.
